# A Scoping Review of Masculinity Norms and Their Interplay With Loneliness and Social Connectedness Among Men in Western Societies

**DOI:** 10.1177/15579883241304585

**Published:** 2024-12-09

**Authors:** Therese Nordin, Frida Degerstedt, Elin Granholm Valmari

**Affiliations:** 1 Community Medicine and Rehabilitation, Umeå University, Umea, Sweden

**Keywords:** men’s health, gender relations, gender equality, social isolation, social participation, identity

## Abstract

This scoping review explores what is currently understood about how masculinity norms interplay with loneliness and social connectedness among men in a Western context. Human beings inherently need social connections, and this review particularly addresses how traditional masculinity norms—emphasizing independence, emotional stoicism, and pain endurance—can increase vulnerability to loneliness and insufficient social connections. The review followed the Joanna Briggs Institute (JBI) methodology for scoping reviews and systematically assessed the literature published between September 2013 and September 2023. Initial literature searches were conducted using multiple databases, including EBSCO and PubMed. Of the 1,730 articles identified, 13 studies met the inclusion criteria and were quality assessed. Results show that men often formed social connections through productive activities. Still, these connections were restricted by conflicting masculine ideals such as self-reliance, which could be prioritized over social and emotional support. Masculine bonds were often formed and strengthened through collectively rejecting diversity. Nevertheless, there were some narratives where participants attempted to redefine masculinity to access permission to care and connect. This review highlights the urgent need for cultural and societal reevaluation of gender norms to support men’s social connectedness. This shift could reduce stigma around emotional vulnerability and in turn men’s opportunities for health and well-being.

## Background

Humans have a fundamental need to create and uphold social connections. When such needs go unmet, loneliness can emerge (Baumeister & Leary, 1995). Loneliness is harmful to physical as well as mental health. The psychological harms include, for example, powerful negative emotions of uselessness (Bindels et al., 2015), shame (Kharicha et al., 2017; Levasseur et al., 2009), and suicide ideation. Loneliness can generates chronic stress, and in turn cause negative cardiovascular (Holt-Lunstad & Smith, 2016) and inflammatory system processes (K. J. Smith et al., 2020). Conversely, individuals with robust social connections tend to experience longer lifespans (Holt-Lunstad et al., 2010, 2015; Holt-Lunstad & Perissinotto, 2023). Hence, social connectedness significantly predicts longevity and better physical, cognitive, and mental health, whereas social isolation and loneliness are significant predictors of poor health and premature death (Holt-Lunstad et al., 2010, 2015; Leigh-Hunt et al., 2017; Luanaigh & Lawlor, 2008; The U.S. Surgeon General, 2023).

There are several ways to describe loneliness. A broad definition includes a painful gap between a person’s desired and actual social connections (Perlman & Peplau, 1981). Some definitions further clarify this discrepancy by distinguishing between different types of loneliness. For instance, “social loneliness” refers to the absence of superficial social activity, such as small talk or help with tasks, while “emotional loneliness” refers to the lack of deeper connections involving trust and emotional freedom (DiTommaso & Spinner, 1997). Another level of loneliness is “existential loneliness,” the feeling of being fundamentally alone and lacking meaning and purpose (Van Tilburg, 2021). All these definitions complement one another, collectively offering a deeper understanding of the complexity of human loneliness and the innate need for social connectedness. Social connectedness, often seen as the opposite of loneliness, is defined as a subjective evaluation of the extent to which one has meaningful, close, and constructive relationships with others—whether with individuals, groups, or society at large (O’Rourke & Sidani, 2017).

Different groups’ shared knowledge, traditions, and ideals deeply affect social connectedness and loneliness, including gendered norms (Connell, 2021; Taylor, 2017). Research by Kågesten et al. (2016) highlights how gendered norms vary significantly across different cultural settings, indicating that masculinity is not a monolithic construct but rather influenced by social, cultural, and economic factors (Kågesten et al., 2016). Masculinity norms have been captured in the Conformity to Masculine Norms Inventory, which includes 11 aspects (winning, emotional control, risk-taking, violence, dominance, playboy, self-reliance, primacy of work, power over women, disdain for homosexuals, and pursuit of status; Mahalik et al., 2005). Such norms prescribe how men should ideally act and behave to be approved by the collective (Connell, 2021). They affect boys from a young age and throughout their lives. The norms may often include encouragement to suppress emotions and expressions coded as feminine, such as empathy, care, and vulnerability (Pollack, 1999), as well as idealizing independence and endurance of physical and psychological pain. These ideals can constrain men’s inclination to disclose problems and to reach out for help because of the risk of penalization in their social groups. Masculinities also differ in different Western contexts, as well as over time. For instance, in Sweden, the notions of masculinity have changed over the last 40 years due to Sweden’s gender equality strategies, where child care has been increasingly included in the masculine ideals (Hearn et al., 2012).

A meta-analysis also found a slight statistical difference suggesting that men were lonelier than women. However, the gender differences varied depending on age and study context (Maes et al., 2019). Regardless, men have been found to have fewer friends (Statistics Sweden, 2023), as well as an increased vulnerability to loneliness (Cox et al., 2020; Franklin et al., 2019; Kealy et al., 2021). The most vulnerable to loneliness are younger men living in individualistic cultures (Barreto et al., 2021). Particularly younger men are at a heightened risk of severe consequences from loneliness, including suicidal ideation (Ernst et al., 2021). However, loneliness is also found in older men, and other social contexts, where social connections are lost, such as unemployment, retirement, or sick leave, which sometimes may influence the decision to end one’s life (Courtenay, 2000; Swedish Association of Local Authorities and Regions, 2018). In summary, men may be more inclined to feel lonely and loneliness is strongly associated with morbidity as well as mortality. As some aspects of masculinity norms may be counter-productive for seeking help and support from others, men may face a normative barrier to forming and maintaining social connections. Despite the known connection between masculinity norms and loneliness, the knowledge is scarce, as to in what way this connection plays. Therefore, this study aims to scope what is currently understood about how masculinity norms interplay with loneliness and social connectedness among men in a Western context.

## Method

A scoping review was considered suitable for exploring knowledge within this subject area, as it constitutes a broad research question that requires mapping and summarizing the evidence (Munn et al., 2018; Tricco et al., 2016). The current scoping review follows the JBI methodology for scoping reviews (Peters et al., 2020).

First, an initial search was conducted in May 2023 to identify the research field and design and refine the search string. Then, a protocol was created and published in OSF, a register for scoping reviews (OSF Center for Open Science, 2024). The protocol (Granholm Valmari et al., 2023) was based on the PRISMA guidelines for scoping reviews (PRISMA-ScR), and the study has been reported in line with the same standards using the PRISMA-ScR checklist (Tricco et al., 2016). One deviation from the protocol has been made, which was adding “social connectedness” to the aim, as it was comprised in the inclusion criteria. This is further discussed in the Strengths and Limitations section. The study did not require ethical approval as it relies on publicly available scientific literature.

### Search Strategy and Study Selection

The initial search was performed in September 2023 by two authors (the first and last authors) separately. The search string was refined based on PIC(o) (Aromataris & Munn, 2020). Boolean strategies “AND” and “OR” were used, together with subject headings in EBSCO databases (APA PsychINFO, Academic Search Premiere, SocINDEX), and MeSH terms in PubMed. See Table 1 for more information on the search strategy, as well as identifying and selecting relevant studies. All search strings created for each database can be found in the protocol (Granholm Valmari et al., 2023).

**Table 1.** Search Strategy, Identifying, and Selecting Relevant Studies

Databases and examples of search string	Study selection criteria regarding population and context
EBSCO databases (APA PsychINFO, Academic Search Premiere, SocINDEX). Truncation was used as well as different subject headings in the databases. Searches were conducted by using author-supplied keywords (KW), subjects (DE), abstract or author-supplied abstracts (AB), and titles (TI), depending on the functions in different databases.	Inclusion criteria • Studies related to masculinity norms and loneliness or social connectedness • Self-identified men • Men in a Western context (Europe, Israel, South and North America, Australia, and New Zealand)
Example search string for APA PsychINFO	
Population and context DE “human males” OR DE “psychologyofmen” OR DE “maleattitudes” OR DE “maleemployees” OR TI “masculinit*” OR AB “masculinit*” OR KW “masculinit*” Phenomena of interest DE loneliness OR DE “social support” ORDE belonging OR DE “social networks” OR DE “social cohesion” OR DE “Social connectedness” OR TI “loneliness” OR TI “lonely” OR TI “social support” OR TI “social cohesion” OR TI “social network*” OR TI “social connectedness” OR TI belonging OR TI “Belong*” OR AB “loneliness” OR AB “lonely” OR AB “social support” OR AB “social cohesion” OR AB “social network*” OR AB “social connectedness” OR AB belong* OR KW “lonel*” OR KW “alone*” OR KW “belong*” OR KW “social participation” OR KW “social isolation” PubMed was searched using the same search blocks, but instead focusing on MeSH terms. The search string was used to search the “titles and abstracts” function.	Exclusion criteria • Participants under the age of 16 years • Studies including both men and women in the results • Severe or chronic illnesses impacting everyday life • COVID isolation studies • Incarcerated or imprisoned men • Gray literature, editorials, psychometric studies, other reviews, or theoretical papers

The current scoping review explored available evidence regarding masculinity norms and loneliness between September 15, 2013 and September 29, 2023. When the searches had been conducted, they were uploaded to RAYYAN, a web application for conducting systematic reviews (Ouzzani et al., 2016). Duplicates were removed by the last author. The first and last authors respectively read titles and abstracts to further narrow the search results following the aim of the study. When uncertainties appeared as to whether to include a study, inclusion was proposed at this stage. Criteria used for inclusion were original papers related to masculinity norms, as well as loneliness or social connectedness, among self-identified men in a Western context (Europe, Israel, South and North America, Australia, and New Zealand). See exclusion criteria in Table 1. Disagreements relating to inclusion or exclusion were discussed between the first and last authors until a consensus was reached.

**Figure 1.**
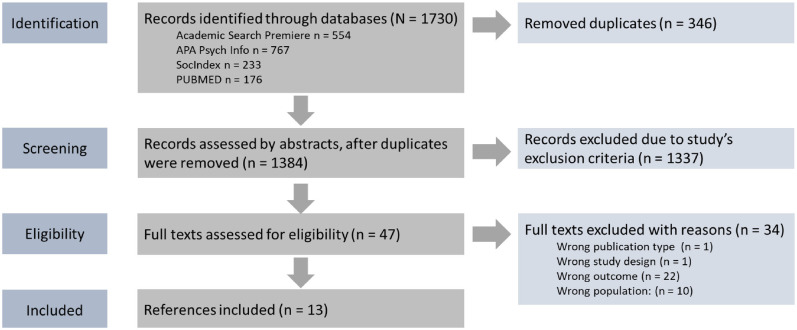
Flowchart According to PRISMA-ScR. Studies That Did Not Match Inclusion Criteria Were Excluded When One Exclusion Criterion Was Met

Ultimately, full texts were read by the first and second authors, and studies not suitable due to the inclusion and exclusion criteria were excluded. Discrepancies were discussed between all three authors resulting in the agreement of the final studies. A flowchart of the entire process, from identifying relevant studies to including the final studies in the review, is presented in Figure 1.

Quality assessment of each study was conducted to inform the readers of potential limitations in the different studies included in our synthesized results, as it is relevant when interpreting the review’s findings (Peters et al., 2020). The included studies were critically appraised by the first and second authors, using the guides “Assessment of studies with qualitative methodology” and “Assessment and studies of exposures” (Swedish Agency for Health Technology Assessment and Assessment of Social Services, 2024). A summary of the quality assessment is presented in Table 1, including a short description of which topics were assessed for the different study designs. The assessment did not affect the inclusion or exclusion of articles in the current scoping review.

Finally, data were extracted using an extraction sheet organized by the second author and reviewed by the last author. The extraction comprised authors, year of publication, country of origin, participant characteristics, methods, theory, and outcome including key findings relevant to our aim. The second author then summarized the studies descriptively (see Table 2). The results of the included studies were analyzed qualitatively by identifying commonalities and differences and sorting them into different topics. The first and last authors synthesized the topics. All authors reviewed and cross-checked the different areas and their content until agreement was obtained.

**Table 2.** Overview of the 13 Articles Included in the Scoping Review

Authors, title, year	Population, country	Aim of study	Study design	Quality assessment^a,b^
Bonnell et al., “There’s no sewing classes, there’s no bedazzling seminars”: The impact of masculinity on social connectedness and mental health for men living in Inner-regional Australia, 2022	29 boys/men, aged 16–73 years 1 nonbinary, 27 heterosexual, 22 partner/married, 8 single/separated, 16 employed full-time, 5 part-time/casual, 5 retired/unemployed, 3 students, 1 no answer, <6 high school, 11 trade/cert/diploma, 4 undergraduate degrees, 9 postgraduate degree Australia	To examine how constructions of masculinity and social connectedness contribute to men’s place-based experiences of mental health in inner-regional Australia	Individual interviews Reflexive thematic analysis Optional photovoice	^a^(+) Only insignificant methodological deficiencies
King et al, “Is life worth living?”: The role of masculinity in the way men aged over 80 talk about living, dying, and suicide, 2020	33 men, age 80–92 years 23 were born in Australia, 6 were born in the United Kingdom, 4 were born in Europe, 1 in another country 14 living alone, 19 living with a partner, 28 living in own home 13 were in very good health, 20 had fair/good health Australia	To understand the role of masculine norms and other life factors in the suicidality of older men	Focus groups Thematic analysis	^a^(+) Recruitment strategy, participants, and theory were acceptably described. Results were validated. (−) Researchers’ reflexivity, background, and relation to participants were insufficiently described.
Lee and Lee, Caring is masculine: Stay-at-home fathers and masculine identity, 2018	25 stay-at-home fathers All married/de facto 18 White, 4 Hispanic/Latino, 1 Black, 1 other 20 college degrees or higher 16 no contribution to the household economy Above average income United States	To understand stay-at-home fathers’ (SAHF) lived experiences focusing on how becoming an SAHF might contribute to shifts in their perceptions of masculinity. Also, examining the challenges of being a male primary caregiver and how SAHFs navigate those challenges	Individual interviews Grounded theory	^a^(+) Recruitment strategy, participants, and theory were acceptably described. (−) Researchers’ reflexivity, background, and relation to participants were insufficiently described. The results were not visibly validated.
McDiarmid et al, “That whole macho male persona thing”: The role of insults in young Australian male friendships, 2017	7 men, aged 22–26 years White Middle class Australia	To explore how young White, heterosexual, working/middle-class men from rural Victoria reflect on their close male friendships in research conversation	Individual interviews Thematic analysis Discourse theory	^a^(+) The researchers’ reflexivity, background, and relation to participants were described. (−) Recruitment strategy, methodology, and theoretical standpoint were insufficiently described, and economic independence was unclear.
McKenzie et al., Social connectedness, and mental health: Men’s diverse patterns of practice, 2018	15 men, aged 20–40 years, Nonimmigrant 14 NZ European, 1 NZ Māori, 14 heterosexuals, 2 Married, 7 de facto, 3 single, 3 divorced, 3 public servants, 1 marketer, 2 law and finance, 1 artist, 2 sports/recreation, 1 engineer, 1 unemployed, 2 university students, 6 prior mental health issues New Zealand	To explore the interplay between masculinity, men’s everyday social practices, mental health, and well-being	Life history method Gender relations theory	^a^(+) Recruitment strategy, participants, and theoretical standpoint are acceptably described. (−) The researchers’ reflexivity, background, and relation to participants were insufficiently described.
Mackenzie et al, Counter and complicit masculine discourse among men’s shed members, 2017	22 men, aged 55+ years 22 White 21 married, 1 widowed ≤12 high school, 10 college/university 1 part-time work, 21 retired/on sick leave Canada	To reflect on how masculinities emerged, as an implicit value, during focus group discussions with older male attendees at a Canadian Men’s Shed	Focus groups The framework method	^a^(+) Recruitment strategy. Participants and theory were acceptably described. Results were validated. (−) Researchers’ reflexivity, background, and relation to participants were insufficiently described. Recruitment inside the program skewed opinions.
Ogrodniczuk et al, Conditional process modeling of the relationship among self-reliance, loneliness, & depressive symptoms, and the moderating effect of feeling understood, 2022	530 men Mean age 48 years Heterosexual 88% Full-time work 55% University degree 44% Canada	To explore the association between self-reliance and depression, while also assessing the roles of loneliness and not feeling understood as contributing factors	Cross-sectional survey study Regression analysis, conditional process modeling	^b^(+) Weighted randomization and stratification (age and religion) were performed to reflect national data. No declared conflicting interests, and no obvious selective reporting. (−) Likely an underrepresentation of highly marginalized individuals without internet access. No defined measure of masculinity.
Ohm and Wechselblatt, Transformative moments among men amid parallel play, 2021	16 men Undergraduates 11 White, 3 Latino, 1 African American, 1 Native American 15 heterosexual, 1 bisexual United States	To explore the complex interplay of personal factors, situational factors, and the presence of cultural barriers that impact the development of close friendships among men, often culturally characterized as bromances	In-depth interviews Exploratory method Grounded theory	^a^(+) The theoretical framework was well described. (−) Inclusion criteria, recruitment strategy, and analysis methodology were not described sufficiently. Researchers’ reflexivity, background, and relation to participants were insufficiently described.
Ratcliffe et al, Reconceptualizing men’s loneliness: An interpretivist interview study of U.K.-based men, 2023	20 men, aged 18–60+ years 12 heterosexual, 1 bisexual 4 Asian, 16 White, 4 South Asian 1 transgender, 19 cisgender 8 solo-living/separated, 5 with partner + children, 2 with a partner no child, 4 with parents, 1 with housemates, 10 higher educated/current student, 10 no higher education United Kingdom	To explore what the influence of gender is on men’s constructions of loneliness, and/or their constructions of their experiences of loneliness	Individual interviews Theoretical thematic analysis Social constructionist epistemology	^a^(+) Only insignificant methodological deficiencies
Ronkainen and Ryba, Is hockey just a game? Contesting meanings of the ice hockey life projects through a career-threatening injury, 2017	2 male ice hockey players, aged 30–40 years Married with children 1 retired 1 professionally active Finland	To analyze two players’ life stories with a focus on injury as a boundary situation involving social and temporal breakdown and reevaluation of the meaning of sporting life projects	Holistic narrative analysis with a critical constructivist approach	^a^(+) Recruitment strategy, participants, and theory were acceptably described as reflexivity and validation of results. (−) Researchers’ relations to participants were insufficiently described.
Willis et al, Loneliness, coping practices and masculinities in later life: Findings from a study of older men living alone in England, 2022	72 men, aged 65–95 years Living alone 51 heterosexual, 21 gay 8 White British, 4 Asian United Kingdom	To examine older men’s responses to feelings of loneliness and their everyday strategies for coping in the context of living alone	Thematic analysis, framework analysis approach	^a^(+) Recruitment strategy, participants, and theory were acceptably described. (−) Researchers’ reflexivity, background, and relation to participants were insufficiently described. Results were not validated.
Willis et al, Loneliness, social dislocation and invisibility experienced by older men who are single or living alone: Accounting for differences across sexual identity and social context, 2022	72 men, aged 65–95 years Living alone 51 heterosexual, 21 gay 68 White British, 4 Asian United Kingdom	To examine how older men who were single and/or living alone talk about loneliness, isolation, and social dislocation, and to identify how differences across sexual identity and social context shape their experiences and observations	Individual interviews Thematic analysis	^a^(+) Recruitment strategy, participants, and theory were acceptably described. (−) Researchers’ reflexivity, background, and relation to participants were insufficiently described. Results were not validated.
Åhlander et al, Living (dys)regulated and alienated young masculinity—Young men’s embodied experiences of mental disorders and suicidality, 2023	13 young men, seeking help for mental illness or suicidal thoughts Age 16–24 years Sweden	To explore young men’s lived embodied experiences of mental disorders and suicidality, and to conceptualize these by integrating affective–emotional, physiological, social, and gendered processes	Individual interviews Grounded theory	^a^(+) Only insignificant methodological deficiencies

a According to criteria in the Swedish Agency for Health Technology Assessment and Assessment of Social Services (SBU) Assessment of studies with qualitative methodology: (a) Coherence between philosophical standpoint/theory, and purpose, selection, and methodology. (b) Participants—description and suitability of selection and recruitment methods. (c) Data collection—potential deficiencies that may affect reliability. (d) The researcher—background and competencies, relation to study participants, reflexivity, and conflicting interests.

b According to criteria in the SBU Assessment of exposure studies: (a) risk of bias from confounders, (b) risk of bias from exposure, (c) dropout, (d) methods to measure outcomes, (e) risk of reporting bias, and (f) conflicting interests.

## Results

This review aimed to scope what is currently understood about how masculinity norms interplay with loneliness and social connectedness among men in a Western context. The separate searches resulted in 1,730 articles (Figure 1). After the removal of duplicates, and reading titles and abstracts, 47 studies were read in full text, of which 34 did not meet inclusion criteria. The final review comprised 13 articles. The publication period was from June 2016 to July 2023. All but one study had qualitative designs. Most concerns regarding the quality of various study designs in the qualitative studies centered around researchers’ reflexivity. In addition, the cross-sectional study had an underrepresentation of a highly marginalized group—specifically, individuals without internet access. Detailed information on quality concerns across the studies is provided in Table 2.

### Review Findings

The findings from the 13 studies are summarized into four topics. The topics revolve around how social connections are pursued through performing productive activities and how self-reliance is a primary ideal that can override social and emotional support needs. Social connectedness was aided by rejecting diversity, but there were also opportunities to transform the view of masculinity to encompass a wider variety of social connections to better fit men’s social needs. The study includes three studies from Australia, two from the United States, one from New Zealand, three from the United Kingdom, one from Finland (conducted in Denmark), and one from Sweden. Altogether, the studies encompass men aged 16 to 92 years, with a total of 326 participants in the qualitative studies (ranging 2–72 participants per study) and 530 participants in the cross-sectional study.

### Social Connections Through Productive Activities

This topic addresses how productive activities such as work or sports provide an arena that allow social connection among men while complying with the restrictions that masculinity norms dictate.

Ratcliffe et al. (2023) describe men’s experiences of loneliness, under the influence of masculine roles and societal expectations. Forming social connectedness was for some facilitated by occupational success or traditionally masculine interests such as sports. Social connectedness strongly interplayed with the men’s sense of respect and gratitude for their role as providers, and if lacking, feelings of disconnection and loneliness emerged. Men’s social connection could also be limited by masculinity norms by not allowing nonmasculine activities. This limited the opportunities for being positively engaged in certain activities.

Social connectedness and masculine norms within the sports community were investigated by Ronkainen and Ryba (2017). Within this context traditionally masculine traits such as strength and pain endurance were emphasized. The team had a strong sense of social connectedness among its members, but when prevented from playing because of injury, the connection was difficult to maintain. Even if players were physically present at games and practices, they experienced being psychologically and socially detached, exacerbating feelings of loneliness. This also seemed to interrupt their sense of belonging and identity. Moreover, King et al. (2020) showed that men’s friends and acquaintances often were work related.

As a consequence of a focus on connections through productivity, life could become empty and lonesome upon retirement, as men’s roles as traditional providers and decision-makers changed (King et al., 2020). One intervention aimed at supporting men’s health was “Men’s sheds” (Mackenzie et al., 2017). The intervention aimed to address men’s loss of relationships and meaningful activity after retirement. Men’s sheds were shown to be a valuable space for reestablishing social connections and reshaping older men’s identities beyond paid labor. The men’s sheds were identified as gendered spaces where older men could connect with other men while reaffirming their identities as men and performing aspects of their masculinity that might be lost in retirement. Doing traditionally masculine activities made it easier to casually connect and initiate conversation with others (Mackenzie et al., 2017). In addition, staying active provided a temporary respite from loneliness, with home-based activities helping to briefly divert attention from isolation (Willis et al., 2022).

### Prioritizing Self-Reliance Over Social Connectedness

This topic addresses how masculinity norms dictate an ideal of being strong, self-reliant, and independent, which harms men’s opportunities to connect with others both socially and emotionally, resulting in feelings of loneliness.

Willis et al. (2022) highlighted the fluctuation between social inertia and proactive efforts to manage loneliness. In attempting to circumvent feeling burdensome or uninteresting to others, the men often avoided asking for help. To bring up loneliness seemed to be particularly difficult because of the stigma and embarrassment associated with loneliness, which further discouraged the men from discussing such problems and feelings. Moreover, Ogrodniczuk et al. (2022) highlighted a complex interplay between self-reliance, loneliness, feeling understood, and depressive symptoms in men’s mental health. Higher levels of self-reliance were associated with increased feelings of loneliness among men and feeling understood moderated the relationship between self-reliance and loneliness. This notion of feeling understood was congruent with the findings of Ratcliffe et al. (2023), where feeling seen and respected was adamant to ward off loneliness. Willis et al. (2022) also described how men felt a strong pressure to uphold an image of independence, keeping loneliness and distress to themselves instead of reaching out to someone. Similar narratives were found in the studies about Men’s sheds (Mackenzie et al., 2017), where the participants for example expressed that the voluntary nature of their participation was crucial for engaging in the provided activities. Adjacent to self-reliance was also the norm of invulnerability.

Ratcliffe et al. (2023) examined men’s willingness to express vulnerability and seek help, and how that, in turn, could affect their mental health and social connections. Hence, being perceived as invulnerable made men reluctant to disclose feelings of loneliness to maintain their image, which made it harder to form close social connections. Moreover, Åhlander et al. (2023) explored the struggles of handling mental health issues within the context of societal expectations. The pressures of adhering to masculine norms discouraged emotional expression. This often led to a lack of communication about their mental health issues and increased their feelings of loneliness. According to King et al. (2020), upkeeping traditional masculine norms, such as being “the strong silent type,” hindered emotional expression and seeking help when needed. Close friendships were not seen as manly and facing bodily decline along with the realization of no longer feeling invincible due to a loss of independence was described as threatening to masculine identity. Feeling loss of independence and being a burden to others were even discussed as valid reasons for suicide.

### Social Connectedness Through Rejecting Diversity and Vulnerability

This topic addresses how avoiding loneliness and maintaining social connectedness were performed through a joint manifestation of masculine ideals that were defined through expressions of rejection of traits that were feminine, nonheterosexual, and non-White.

McDiarmid et al. (2017) showed how young Australian men used derogative language as a communicative strategy to convey intimacy and reinforce social connectedness, but also to assert dominance. For example, insulting bantering seemed to mirror traditional masculine ideals. To degrade friends by rejecting feminine, nonheterosexual, and non-White was also congruent with how Mackenzie et al. (2017) described “male-friendly banter” as an important way of connecting socially with other men. Masculine activities and needs were primarily constructed through the rejection of nonmasculine traits, and men’s needs to share emotions and connect were constructed as contradictory to the way women connect. To be the right kind of man: being non-feminine, heterosexual, and White were presented as a prerequisite to be included in the male community (Mackenzie et al., 2017). Another communicative strategy to socially connect and convey support was to intentionally use silence. By avoiding bringing up sensitive issues, they could convey concern or gratitude to their friend without risking revealing potentially nonmasculine emotions. Furthermore, interrogations could be used strategically to signify a serious mood when discussing sensitive issues (McDiarmid et al., 2017). Bonell et al. (2022) describe similar patterns in their study of masculinity and social connectedness in regional Australian settings. Men who deviated from traditional masculinities felt pressured to conform to strong masculine ideals in other ways, such as being interested in guns and alcohol and normalizing sexism and homophobia. Failing to conform to these ideals entailed the risk of exclusion from the local social community. Meanwhile, people immigrating to the regional area from the city could challenge these rigid ideals by contributing to alternative ways of performing masculinity.

### Reinventing Masculinity to Access Social Connectedness

This topic addresses how reinventing masculinity could fulfill social needs by challenging traditional norms around emotional expression and social connections. To transform their role experiences redefined masculinity and allowed greater emotional openness and support.

According to King et al. (2020), close friendships were generally seen as unmanly. However, McKenzie et al. (2018) found that men often separated their social connections between those with other men and those with women. Relationships with men tended to be more activity-based and less emotionally open, while those with women were more likely to involve connecting on a deeper level with space for emotional support. This division allowed men to adhere to traditional masculine norms in public while still accessing intimate connectedness and support in private. However, some men experienced transformative relationships that challenged traditional norms, often triggered by significant life events that pushed them to seek deeper social connections (McKenzie et al., 2018). Reaching out to family or people in their vicinity was also important to mitigate loneliness among older men, according to Willis et al. (2022).

Ohm and Wechselblatt (2021) explored how deep male friendships could challenge traditional masculinity norms. The participating men described how meeting their needs for connection and support was difficult under the present expectations of suppressing emotion and being independent. However, competence in articulating emotions was helpful when navigating the demands of masculinity norms and could help deepen relationships. Instances of planned or coincidental connectedness with other men could be transformative, offering new perspectives on masculinity and emotional connections that defied societal expectations. The study by Lee and Lee (2018) with stay-at-home fathers also showed how men’s views on traditional masculinity concerning full-time child care sometimes clashed with their masculine identity. This could ultimately lead to evolved views on masculinity encompassing softness, closeness, and responsiveness. Moreover, this transformed view could sometimes be relabeled to fit into other traditionally masculine ideals, such as being a family man, or be discarded altogether and replaced by other, more flexible, values. However, fulfilling some aspects of traditional masculinity, for example, being strong, made it easier to break other norms, such as playing dress-up, without losing their identity as men. Ratcliffe et al. (2023) also describe how rejecting dominant masculinity norms, for example, through expressing vulnerability or living openly gay, partly alleviated loneliness.

## Discussion

### Result discussion

This review synthesizes key insights into how masculinity norms interplay with loneliness and social connectedness among Western men. The main findings reveal how productive activities could provide an arena for social relationships, but at the same time, restrict the prerequisite for social connectedness. Self-reliance could be prioritized over social connectedness, and rejecting traits that were feminine, nonheterosexual, and non-White was sometimes used to connect with other men. However, the men also redefined masculinity to access social connectedness, challenging traditional norms through a transformative process of roles.

The findings from this review show that rejecting homosexual or racialized men is a way of connecting socially by creating “us” and “them.” This is in line with Van Vugt et al. (2007) who suggest that men’s cooperation with each other is regarded to be the strongest during competition against an external group. Hence, without sharing a common goal, it might become more difficult for men to socially connect without also downplaying their masculinity. This could potentially lead to loneliness. If it, despite efforts, was too difficult to maintain needed social roles within masculinity, there were instances where the men challenged traditional masculinity and adopted more emotionally open roles. Transforming their masculine norms offered opportunities for men to maintain their masculine identity while forming deeper connections. Such a process is, according to Dufault and Meunier (2017), influenced by various social, economic, and cultural factors, leading to a reevaluation of traditional masculine identities and practices. However, the reinvention of masculinity norms is a multifaceted process including an overall societal process that values emotional connectivity and relational engagement (Dufault & Meunier, 2017).

We also identified that masculine communication styles—such as banter and insults—are used to socially connect with other men, as well as to avoid loneliness. While these interactions may reflect trust among well-acquainted men, they also perpetuate the rejection of femininity, aligning with Connell (2021). Moreover, restricting emotional expression, as our study shows, impedes close friendships, leading to emotional loneliness (DiTommaso & Spinner, 1997), which is detrimental to health. In addition, men in our study were found to compartmentalize their social relationships, with female relationships offering deeper emotional support without threatening their masculine identity. This is in line with previous research on male loneliness, where it has been connected to men’s reluctance to seek help for emotional and mental health-related issues (Cox et al., 2020; Kealy et al., 2021).

Our results illustrate how masculine ideals, such as self-reliance, strength, and emotional suppression, can limit social connectedness. Engaging in traditionally masculine activities, such as sports or work, offers men a way to connect without compromising their masculine identity. These arenas can become more restrictive when productivity is limited due to factors like injury or retirement, leaving them vulnerable to loneliness. In line with Barreto et al. (2022), men also tend to experience more shame and rejection because of emotional problems and loneliness than women (Barreto et al., 2022). This may be a result of the expectations of autonomy and strength. Also, due to the masculine construct of self-reliance discouraging help-seeking, research has shown that men often resort to counter-productive coping strategies such as substance abuse, risky behavior, social isolation, or prolonged work hours instead of seeking assistance. In some cases, this even results in suicide (Seidler et al., 2016). Hence, our results are in line with Courtenay (2000) who shows how gender roles shape social relationships, as well as with research by Seidler et al. (2016) on masculinity’s influence on help-seeking behavior.

Finally, our findings highlight the need to address masculine norms in interventions designed to reduce loneliness. This aligns with Jones et al. (2023), who found that certain attitudes toward masculinity and mental health deterred some men from discussing their mental health concerns. This, in turn, increased their loneliness. Therefore, to promote social connectivity among men, interventions should focus on fostering a sense of belonging within men’s family, work, and community environments, while also creating spaces for help-seeking that allows preserving masculine identity (Keohane & Richardson, 2018). Examples from previous studies include health promotion interventions such as barbershops (Hood et al., 2018), a coffee group providing social support for older men (Broughton et al., 2016), and fraternities for young men (McGuire et al., 2020). Encouraging action on social determinants of health is thus essential for men’s health (J.A. Smith et al., 2020).

#### Strengths and Limitations

The results of the current scoping review should be interpreted in the context of its identified limitations. The search strategy primarily identified qualitative studies, which entails both potential strengths and limitations. The benefits of qualitative research include the potential for capturing the complexity of phenomena and gaining insights into human experiences. In addition, summarizing and abstracting qualitative data allow contrasting and comparison across studies. However, in this process, there is also a risk of misrepresenting participants’ perspectives. Readers of qualitative research should always be cautious when considering the transferability of qualitative findings. Another limitation is the small number of included studies, most of which employ qualitative designs. This might have been a result of our exclusion criteria focusing on findings only including men, or on the search string mainly searching for aspects related to masculinity. However, the narrow scope of social connectedness and loneliness specifically with masculinity norms also made the results tangible and facilitated synthesis, which should be considered a strength of the review. In addition, only peer-reviewed research articles were included, which means gray literature was excluded. The complex relationship between masculinity norms, loneliness, and social connectedness cannot be fully captured by this review alone. However, the findings still provide a valuable foundation for further research on the subject.

The scoping review focuses exclusively on Western men. While loneliness might be significant issues for men across all countries and cultures, we chose to concentrate on men in Western contexts. We recognize the diversity of cultures and subcultures within Western societies and acknowledge that the potential meanings of these different subcultures have not been addressed in this study. In addition, masculinities vary across Western countries. These perspectives merit further investigation, and this scoping review may serve as a foundation for future research on masculinities within Western cultures, which are generally viewed as more individualistic. This has been suggested to impose a greater emotional burden compared to more collectivist Eastern cultures (Mastracci & Adams, 2019). Our cultural focus limits the applicability of the findings to non-Western contexts.

A further limitation involves a smaller deviation from the original review protocol (Granholm Valmari et al., 2023). The aim was clarified to suit the search strategy used. Hence, social connectedness was added to the aim of the study, as it emerged as an important factor for finding studies to include related to loneliness, leading to its inclusion in the review. This adjustment was made to enhance understanding of men’s loneliness and social relationships, as social connectedness represents the converse of loneliness and offers a more comprehensive view of the relationship with masculinity norms. Despite these limitations, we adhered closely to the guidelines set by the JBI Manual for Evidence Synthesis (Peters et al., 2020) in planning and conducting this scoping review. In addition, we chose to assess the quality of the studies included, which strengthens the reliability of our findings and should be considered a strength of this review.

## Conclusion

The findings of this scoping review highlight the complex relationship between masculinity norms and men’s loneliness and social connectedness. The review shows that traditional masculinity norms may limit men’s ability to form meaningful social connections by prioritizing self-reliance and invulnerability over emotional expression and support. While work and sports provide opportunities for connection, they can also reinforce alienating norms, especially in times of transition like retirement or injury. The rejection of nonmasculine traits further strengthens exclusionary behaviors. However, there are opportunities for change. Redefining masculinity through life events, deeper friendships, or new roles can challenge these norms and foster social connectedness, hence avoiding loneliness.
